# Proposal of Mapping Digital Twins Definition Language to Open Platform Communications Unified Architecture

**DOI:** 10.3390/s23042349

**Published:** 2023-02-20

**Authors:** Salvatore Cavalieri, Salvatore Gambadoro

**Affiliations:** Department of Electrical Electronic and Computer Engineering, University of Catania, Viale A. Doria 6, 95125 Catania, Italy

**Keywords:** digital twin, Industry 4.0, DTDL, OPC UA, interoperability

## Abstract

The concept of Digital Twin is of fundamental importance to meet the main requirements of Industry 4.0. Among the standards currently available to realize Digital Twins there is the Digital Twins Definition Language. Digital Twin requires exchange of data with the real system it models and with other applications that use the digital replica of the system. In the context of Industry 4.0, a reference standard for an interoperable exchange of information between applications, is Open Platform Communications Unified Architecture. The authors believe that interoperability between Digital Twins and Open Platform Communications Unified Architectures communication standard should be enabled. For this reason, the main goal of this paper is to allow a Digital Twin based on the Digital Twins Definition Language to exchange data with any applications compliant to the Open Platform Communications Unified Architecture. A proposal about the mapping from Digital Twins Definition Language to the Open Platform Communications Unified Architecture will be presented. In order to verify the feasibility of the proposal, an implementation has been made by the authors, and its description will be introduced in the paper. Furthermore, the main results of the validation process accomplished on the basis of this implementation will be given.

## 1. Introduction

The fourth industrial revolution (Industry 4.0) has been aimed from its very beginning to the creation of more and more flexible, interoperable and innovative systems and services to achieve effective business models that enhance the quality of production [1,2,3].

Industry 4.0 is featured by a continuously-evolving digital transformation. It aims to automate all the traditional industrial practices, and it hopes to do so by bringing as much of the equipment from the physical space into the virtual domain. This is where the Digital Twin (DT) comes into play. Digital Twin emerged as an experimental technology set to enable replication of elements, functions, operations and dynamics of physical systems into digital world, with better control at testing, analysis, prediction and hazard prevention for sensitive processes [4,5].

Digital Twins are used in different industrial settings, including health surveillance, agriculture, smart cities, smart grids, manufacturing, meteorology, education and automobiles. Digital Twins support the development of production processes making them reliable and flexible, enabling to visualise, monitor and optimize processes [4,5,6,7].

Different organisations are currently working aiming to standardize Digital Twin definition, interoperability and how to interact with these Digital Twins. Two notable projects in this area are the Asset Administration Shell (AAS) [8,9,10] and the Digital Twin Definition Language (DTDL). The DTDL was born as an open-source initiative by Microsoft, and it is already used in many commercial services offered by Microsoft, such as IoT Hub, IoT Central and Azure Digital Twins [10,11].

One of the main features of a Digital Twin is the communication with the physical world, from which a Digital Twin must receive the current state (e.g., collection of data measured by sensors). At the same time a Digital Twin has the need to publish output data produced as a result of the processes accomplished on the physical system for which it realizes a replica. A Digital Twin may have also the need to exchange data with different applications according to the aims to be reached (e.g., monitoring, testing, analysis, prediction and maintenance). For this reason, data exchange must be considered an important part of a Digital Twin; without data exchange, most of functions of a Digital Twin could not be realized [12]. Interoperability of the data exchange seems a very important requirement, allowing a Digital Twin to communicate with a multitude of physical systems and applications.

Indeed, in the context of Industry 4.0, much effort has been spent and is currently being accomplished to fully integrate industrial applications in a multitude of production-related fields, safety and communication of machines, where they must interact and align their information models. An overview of the main trends about data integration and interoperability may be achieved by reading [13,14]. Interoperability between data and applications in industrial context is considered one of the main goals of Industry 4.0 [15,16,17].

Open Platform Communications Unified Architecture (OPC UA) [18], is considered one of the main reference standards for an interoperable exchange of information between applications inside Industry 4.0 [19]. OPC UA is widely used in industry as it is regarded as the most accepted protocol that harmonizes machine-to-machine interaction [20]. On the basis of its powerful capabilities, OPC UA is one of the main candidates for leading the standardization and systems integration for present and future application frameworks [19,21]. The OPC UA is based on two communication models: client/server and publish/subscribe; a comprehensive information model allows to represent data and the relevant semantics.

**Figure 1:**
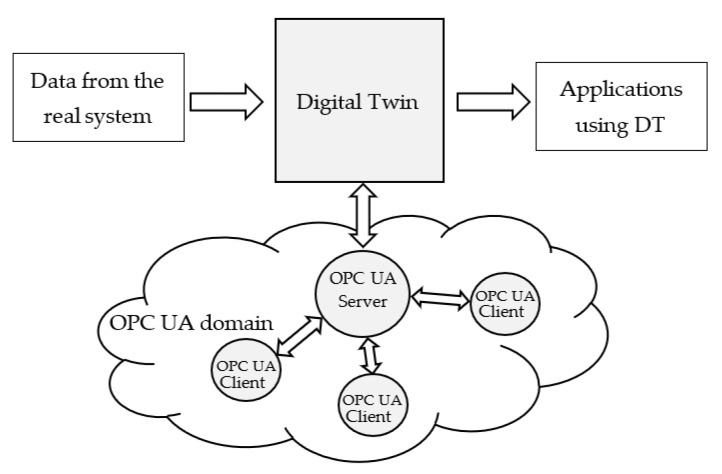
Graphical representation of the proposal.

The idea behind the paper is to enhance the interoperability of a Digital Twin through integration into the OPC UA domain. Figure 1 shows a Digital Twin exchanging data with the real system it models, and with applications using the DT (as said before, these applications may realize data analytics or maintenance, for example). Data exchange with applications based on OPC UA communication system may be enabled through a solution able to map the entire set of information maintained by a Digital Twin into the OPC UA domain. Mapping should include every semantic aspect of the Digital Twin, in order to really enable an interoperable data exchange between the two domains. The proposal presented in this paper is based on the use of an OPC UA Server able to realize this mapping. The OPC UA Server shown by Figure 1 is able to represent each element of the Digital Twin in the OPC UA domain, making available the Digital Twin and its relevant content to whatever application based on OPC UA client role. The mapping solution here presented enables a Digital Twin to have a counterpart in the OPC UA domain; each information (including semantic) maintained in a Digital Twin can be published by the OPC UA Server, allowing an interoperable data exchange with a plethora of OPC UA-compliant applications.

Among the available Digital Twins, the Digital Twin Definition Language model will be considered in this proposal. The paper will present the use of the OPC UA information model to structure and display a Digital Twin based on DTDL. The definition of a custom data structures in the OPC UA information model, able to represent each element of the original DTDL-based Digital Twin, will be introduced in this work. The proposed mapping has been implemented in order to be validated; the relevant implementation and validation will be introduced in the paper.

The paper is organised as it follows. Section 2 will highlight the related work in literature, pointing out the originality of this proposal. Section 3 and Section 4 will give an overview on OPC UA and DTDL, respectively. Section 5 will describe the proposed mapping between DTDL and OPC UA information model. Section 6 will describe the implementation of the proposed solution and the process adopted for the relevant validation. A final section will allow to make discussions about the proposal and conclusions.

## 2. Related Work

As said in the introduction, the main feature of the proposal is the use of OPC UA information model to realize a mapping from the representation of the data exposed by a DTDL-based Digital Twin and the OPC UA domain. Given this goal, this section aims to analyze the works present in the current literature in order to deal with the two important following issues.

The use of OPC UA in this proposal is justified if the reader realizes that OPC UA is widely used in the current literature to realize mappings of information model between different domains; for this reason, the first part of this section is devoted to this demonstration giving to the reader the overview of the main publications related to the use of OPC UA to the mapping of different domains.

The proposal of using OPC UA to map the DTDL-based Digital Twin, is justified if current literature does not present any other work about the same subject; for this reason, the second part of this section is devoted to this demonstration, giving an overview about the entire set of publications about the integration of OPC UA and the DTDL-based Digital Twin.

Current literature provides a lot of publications pointing out the advantage in terms of interoperability of the use of OPC UA information model to structure and expose data coming from different domains of interest. An overview of examples of integration of OPC UA and other domains currently present in the literature, will be given in the following. In [22] a proposal to map Common Industrial Protocol (CIP) to OPC UA is given; as known, CIP is an industrial protocol for industrial automation applications and the proposal of a mapping with OPC UA is aimed to enhance its interoperability. In [23], mapping of EtherCAT communication standard to OPC UA is presented; in this paper is clearly stated that OPC UA is a widely used industrial communication middleware and ensures interoperability between the devices, offering the possibility to map domain-specific data models to OPC UA information model. In [24,25], interoperability between OPC UA and oneM2M is dealt; this mapping allows the enhancement of interoperability with Internet of Thing (IoT) domain, as oneM2M is considering one of the leading standards for the IoT. In [26,27], the mapping between the OPC UA and OCF computer model is presented, enabling interoperability in the smart home and smart building domain. In [28], mapping between OPC UA and the IEC 61804 Electronic Device Description Language (EDDL) is presented; this work allows integration of field device descriptions based on the international standard IEC 61804 into OPC UA. Another example is given by [29] where mapping between OPC UA and Unified Modeling Language (UML) is presented. A formal mapping between OPC UA and the Semantic Web is given by [30]; an OPC UA-based mapping solution for statistically and dynamically typed programming languages is proposed in [31]. A paper [32] presented an interoperability solution based on OPC UA in service-oriented architectures. A mapping of OPC UA with IEC 61850 SCL SmartGrid was proposed by [33].

The overview just given demonstrates that OPC UA is widely used to map different domains enhancing interoperability. One question remains unanswered; it is about the existence of mapping approaches between OPC UA and DTDL-based Digital Twins. This question is very important in order to point out the originality of the paper. For this reason, the second step accomplished in this section is that to give to the reader an overview about the entire set of publications about the integration of OPC UA and the Digital Twins and in particular about DTDL-based Digital Twins.

Among the Digital Twins currently available, several works focus on the mapping of Asset Administration Shell DT and OPC UA. Mapping of AAS DT to OPC UA is proposed by [34]. Another example of integration of AAS with OPC UA is given by [35]. Integration of AAS DT and OPC UA is also subject of the official specifications [8,36], which define an OPC UA model to expose AAS information to OPC UA applications and to exchange asset information between Industry 4.0 components.

To the best of authors’ knowledge, considering the integration of DTDL-based Digital Twin with OPC UA, only the software solution, called OPCUA2DTDL [37], is currently available. This solution aims to convert an OPC UA information model into DTDL constructs; a DTDL digital model can be built starting from its definition based on OPC UA specification. The main limit of this solution is that mapping in the opposite direction is not allowed. In other terms, given an already defined DTDL digital twin is not possible to achieve its counterpart in the OPC UA domain. Introduction pointed out that the paper has this aim, proposing an integration able to map a Digital Twin realised by DTDL into OPC UA information model. As Figure 1 shows, the proposed mapping solution is able to represent every data collected by a DTDL-based Digital Twin into the OPC UA domain; in other words, the mapping proceeds from the DTDL-based Digital Twin towards the OPC UA and not in the other way.

In conclusion, this section pointed out that OPC UA is one of the main standards used to enhance interoperability of different domains by mapping solutions, and to the best of authors’ knowledge, the proposed mapping solution between DTDL and OPC UA is original.

## 3. OPC UA Information Model

The OPC UA communication standard is based on both client/server and publish/subscribe communication models and provides a semantically enriched information model in order to represent data.

The OPC UA Information Model provides a standard way for servers to expose information to clients; publish/subscribe communication model is based on the same information model used for the client/server communication model. The set of information is maintained through OPC UA Nodes grouped together to compose the so-called OPC UA AddressSpace [18,38,39]. The OPC UA Information Model is based on object-oriented programming, so that some nodes representing instances inherit from other nodes defining types; multiple inheritance and object composition are allowed [38].

Each OPC UA Node belongs to a class named NodeClass. Each NodeClass is derived from the Base NodeClass which defines the common attributes of OPC UA Nodes, among which: NodeId (which unambiguously identifies a Node in the OPC UA AddressSpace), Description (which is a textual description of the OPC UA Node), BrowseName (used to identify the OPC UA Node when browsing the OPC UA AddressSpace), WriteMask (exposing the possibilities of a client to write the attributes of the Node), NodeClass (which identifies the NodeClass of a Node) and DisplayName (which contains the name of the OPC UA Node that should be displayed in an user interface). In the following, a very concise overview of the main OPC UA NodeClasses will be given.

Variable NodeClass is used to model data. It features an attribute named Value, containing the data, and an attribute named DataType specifying the type of the content of the attribute Value. Two types of Variables are defined: Property and DataVariable. Property contains information describing particular features of other OPC UA Nodes (e.g., semantic information). A DataVariable maintains data values of a particular system (e.g., information produced by a temperature sensor in a control system).

Method NodeClass allows to model callable functions that initiate actions within an OPC UA Server. One Method features two OPC UA Properties named InputArguments and OutputArguments, used to specify the input and output arguments of the Method.

Object NodeClass is used to represent real-world entities such as system components, hardware and software components or even a whole system. An OPC UA Object is a container for other OPC UA Objects, Variables and Methods. As the Object Node does not provide for a value, an OPC UA DataVariable Node must be used to represent the data of an Object.

OPC UA Information Model includes NodeClasses defining types. ObjectType NodeClass is used to hold type definition for OPC UA Objects. OPC UA defines the BaseObjectType which all the ObjectTypes must be extended from. OPC UA already defines several standard ObjectTypes derived from BaseObjectType. An example is FolderType whose instance, named Folder, is an Object organizing the AddressSpace into a hierarchy of OPC UA Nodes; it represents the root node of a subtree.

VariableType NodeClass is used to provide for type definition of Variables. OPC UA standard defines the BaseVariableType, which all the VariableTypes must be extended from. Moreover, several standard VariableTypes derived from BaseVariableType are already defined by the standard. Among them there are the BaseDataVariableType and the PropertyType. The former is used to create an instance of a DataVariable Node, whilst the latter defines a Property Node.

DataType NodeClass is used to provide type definition of the Value attribute of a Variable Node, as said before. DataType may be Built-in, Enumeration or Structured; arrays of elements are also allowed.

Relationships may be defined between OPC UA Nodes; they are called References. The ReferenceType NodeClass is used to define different semantics for References. References may be classified in two different main categories: Hierarchical and NonHierarchical.

Among the Hierarchical References, the following ones will be used in the paper: HasComponent, HasProperty and Organizes. The HasComponent is a Reference featuring an OPC UA Object or DataVariable as the source, and an OPC UA Object, a DataVariable or a Method as the target Node. If the source is an Object, the semantic associated to this reference is that the source is made up by the target OPC UA Object, DataVariable and Method Node. If the source OPC UA Node is a DataVariable, the target Nodes must be other OPC UA DataVariables; the meaning, in this case, is that the source variable is made up by a set of other variables. HasProperty Reference may connect a source OPC UA Node to an OPC UA Property Node; the meaning is that the source Node features a property described by the target Node. Organizes Reference allows to organize OPC UA Nodes inside a Folder.

Among the NonHierarchical References, there are the HasTypeDefinition, HasSubtype and HasModellingRule. HasTypeDefinition is used to bind an OPC UA Object or Variable to its ObjectType or VariableType, respectively. HasSubtype Reference expresses a subtype relationship between types. The HasModellingRule Reference is used to describe how instances of a particular type should be created. The source of this Reference is named InstanceDeclaration, whilst its target is a ModellingRule Object. An InstanceDeclaration is an Object, Variable or Method that is the target of a Hierarchical Reference starting from an ObjectType or VariableType Node (each of which will be called OPC UA type in the following). A ModellingRule Object specifies what happens to the InstanceDeclaration with respect to instances of the relevant OPC UA type. Several ModellingRule Objects are defined in OPC UA. A Mandatory ModellingRule for a specific InstanceDeclaration specifies that instances of the OPC UA type referencing the InstanceDeclaration must have a counterpart of that InstanceDeclaration. This means that each instance must hold an OPC UA Node of the same NodeClass of the InstanceDeclaration; furthermore, the Reference targeting this OPC UA Node must be of the same ReferenceType of the one pointing the InstanceDeclaration. An Optional ModellingRule for a specific InstanceDeclaration, instead, specifies that instances of the OPC UA type may have a counterpart of that InstanceDeclaration, but it is not required. Other two ModellingRule Objects exist, named MandatoryPlaceholder and OptionalPlaceholder. The difference with the previous ones is that the counterparts of InstanceDeclaration may be more than one.

OPC UA defines standard graphical representation for both Nodes and References. Some of them are summarized by Table 1 and Table 2, respectively. For more information, please refer to Annex C of [38].

**Figure 2:**
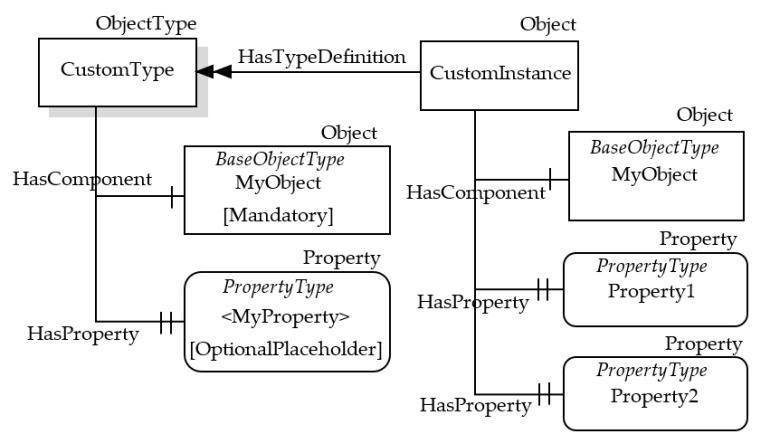
Example of OPC UA graphical representation.

Figure 2 shows an example of the graphical notation just described. An OPC UA ObjectType named CustomType is described; it is the source of an HasComponent Reference targeting the InstanceDeclaration MyObject, and it is the source of HasProperty Reference targeting the InstanceDeclaration <MyProperty>. These two InstanceDeclarations target a Mandatory ModellingRule Object and an OptionalPlaceHolder ModellingRule Object, respectively. As shown by Table 1, these ModellingRule Objects and the relevant HasModellingRule References are not represented, limiting the graphical representation to the texts [Mandatory] and [OptionalPlaceholder] inside the InstanceDeclarations.

In order to better understand the role of the InstanceDeclaration and ModellingRule Object, Figure 2 shows, on the right, a possible instance of the CustomType ObjectType, called CustomInstance. It features a HasComponent Reference targeting the Object MyObject, which is the counterpart of the InstanceDeclaration with the same name. CustomInstance Object features two HasProperty References, used to target counterparts of the InstanceDeclaration <MyProperty>. As this Property points to an OptionalPlaceholder ModellingRule Object, more than one instance may be present; in this example, two instances have been considered, with two different Names (i.e., Property1 and Property2) are shown by Figure 2.

**Figure 3:**
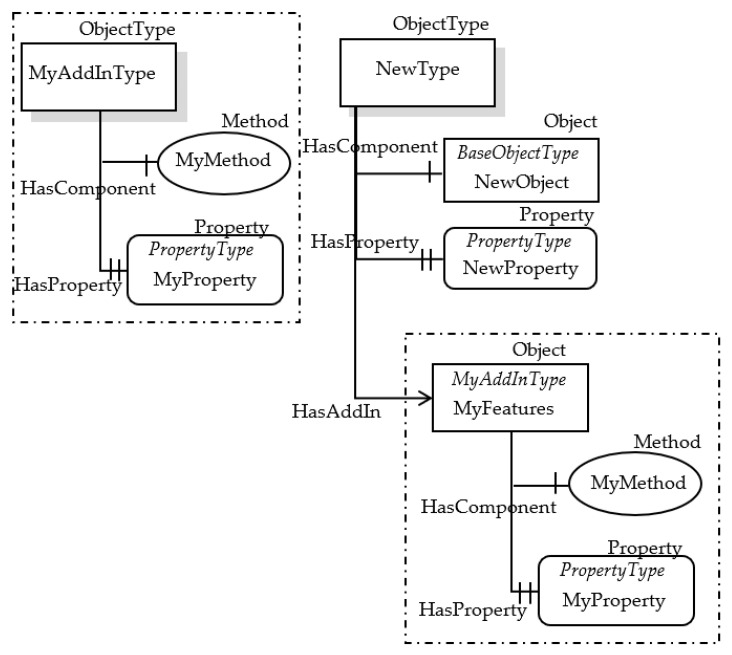
Example of OPC UA AddIn and HasAddIn Reference.

Very recently, a new feature has been added to the OPC UA Information Model, called AddIn [38,39]; it is based on the idea of object composition. An AddIn is an Object that adds features (represented by its ObjectType) to the Node it is applied to. An AddIn is applied to a Node by adding a Reference pointing to the AddIn Instance; a HasAddIn Reference or a subtype shall be used. There are no restrictions for AddIn ObjectTypes and there is no special super type for AddIns. The OPC UA feature based on AddIn and HasAddIn allows information modelers to create re-usable components that may belong to many different ObjectTypes. Figure 3 shows an example of AddIn and HasAddIn Reference.

In Figure 3, the AddIn Object called MyFeatures is added to the ObjectType NewType (and to all the relevant instances, i.e., Objects of type NewType). The features added through the AddIn are relevant to the MyAddInType ObjectType shown in the same figure, on the left.

## 4. Digital Twins Definition Language

The Digital Twins Definition Language (DTDL) [11] was born from a Microsoft initiative, with the aim of implementing a language capable of describing Digital Twin devices and Digital Twin assets. For the creation of the DTDL, Microsoft relied on JSON-LD, a connected data exchange format using JSON [40]. DTDL uses a structure based on classes and metamodels, very similar to the Asset Administration Shell [8].

The DTDL implements six classes of metamodels: Interface, Telemetry, Property, Control, Relationship and Component. These classes are able to fully define the structure and behaviour of a Digital Twin.

In DTDL, resources are called interfaces and can contain a set of telemetry, properties, commands, relationship and components. Telemetry describes data emitted by a resource, whether it is a regular stream of sensor readings or a calculated data stream, such as the raw data of sensors, processed data or data generated by DT models; Telemetry does not store any data. Properties define values within a Digital Twin; these values can be read-only or have read and write states. Properties have a backing storage; this allows us to read the value of the property at any time. However, the property can also be writable according to a particular setting; this allows us to store a value in the property.

Commands correspond to functions that can be invoked with optional input and output parameters. Relationship describes a link to another digital twin and makes it possible to create graphs of digital twins. Component models the entities that exist in the DT, including sensors, gateways, and digital systems.

## 5. Mapping Solution from DTDL to OPC UA

This paper aims to present a proposal to map the DTDL metamodel classes (i.e., Interface, Telemetry, Property, Command, Relationship and Component) into corresponding elements in the OPC UA Information Model.

As it was pointed out in Section 2, literature provides a lot of publications featuring the use of OPC UA Information Model to structure and expose information coming from different domains of interest. In general, the procedure used to reach this aim requires a phase where all the requirements of the original domain of interest are collected and compared with the standard elements of the OPC UA Information Model in order to find the best mapping between them. Often, this is not an easy task because some concepts from the source domain cannot be directly mapped into OPC UA; in these cases, the definition of new types extending the original OPC UA elements must be realized. The reader may refer to [34] to have an overview of the common practices adopted when OPC UA Information Model is used to model a generic system.

In this proposal, the above-mentioned common practices have been taken into account to map the DTDL metamodel classes into OPC UA Information Model; custom OPC UA types have been defined through an extension of the current OPC UA Information Model, able to represents the DTDL metamodel classes and the relevant properties. The following subsections will present the mapping of the six DTDL metamodels classes, introducing the custom OPC UA types defined to realize the proposal.

### 5.1. Interface

The metamodel class Interface allows to describe the contents of a digital twin, as told in Section 4. Interfaces are reusable and can be reused as components in another Interface. Table 3 shows the properties defined for this class.

It has been assumed to map the Interface metamodel class with a custom OPC UA ObjectType, called DTDLInterfaceType. The DTDLInterfaceType will inherit all the attributes of the BaseObjectType, including those of the OPC UA Base NodeClass. Some of these last attributes are used to represent a subset of the properties of the Interface class. Table 4 gives the mapping of the properties of the Interface class with the attributes of OPC UA DTDLInterfaceType ObjectType.

The DTDL @id property is used to univocally identify each metamodel class. On the basis of the definition of NodeId given in Section 3, it is clear that this property may be represented by the NodeId attribute of the OPC UA Node used to map the metamodel class. The DTDL displayName and description properties give a localized name and description of the interface, respectively; they may be represented by the OPC UA DisplayName and Description attributes, respectively, as they share the same meaning.

Table 3 shows the presence of other properties, which have been mapped, as it will be explained in the following.

In DTDL, the @type property is used to specify the type of the metamodel class; in this case, this property must be set to the “Interface” value. As said in Section 3, each OPC UA Node features the HasTypeDefinition Reference which allows to bind an Object or Variable to its ObjectType or VariableType, respectively. For this reason, the DTDL @type property may be represented by the HasTypeDefinition Reference pointing to the OPC UA DTDLInterfaceType type for each OPC UA Object used to represent the DTDL Interface. The DTDL @context property is used to specify the version of DTDL being used when writing a digital twin definition. This information has no counterpart in the OPC UA domain, and for this reason, its mapping is not required.

**Figure 4:**
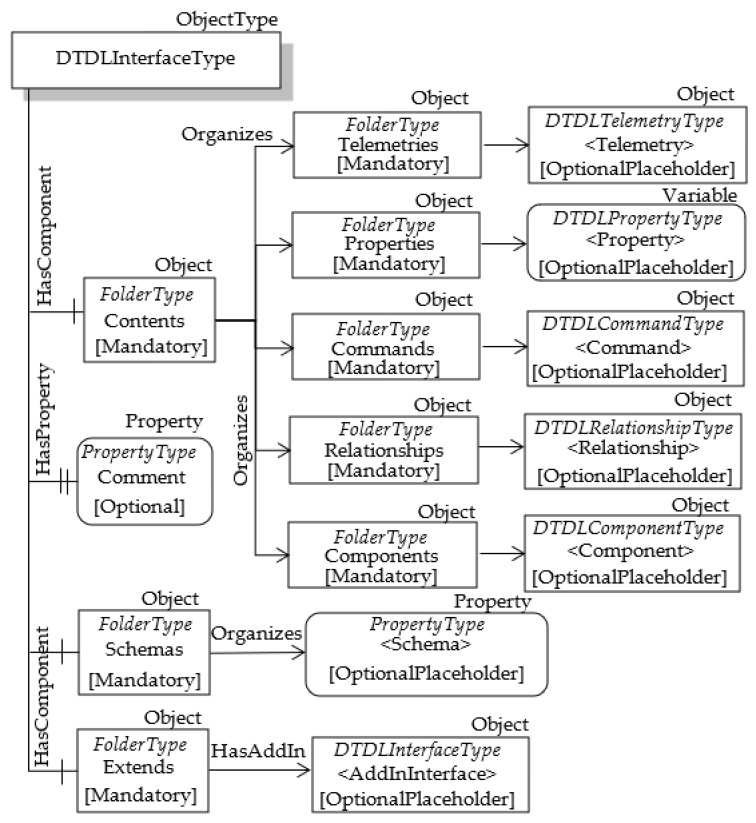
OPC UA DTDLInterfaceType.

The remaining properties of Interface class shown by Table 3 (i.e., contents, comment, schemas and extends) were mapped using OPC UA Nodes connected to the OPC UA DTDLInterfaceType ObjectType, as shown by Figure 4.

The DTDL contents property is very important as it allows to define the set of objects representing the contents of an Interface; these objects belong to the other DTDL metamodel classes, i.e., Telemetry, Property, Command, Relationship and Component. As suggested in [34], an element representing a set may be mapped into OPC UA using a folder, i.e., an Object of FolderType; this folder will organize the elements contained in the set. For this reason, the mandatory FolderType Object called Contents is present in Figure 4. As the DTDL contents property allows the definition of a set of different elements of different types, in the proposal it is suggested to organize the different types of elements using other folders. For this reason, Figure 4 shows the presence of other mandatory folders, called Telemetries, Properties, Commands, Relationships and Components. Each of this folder will organize the OPC UA Nodes used to represent the other DTDL metamodel classes, i.e., Telemetry, Property, Command, Relationship and Component; the relevant mapping will be described in the following subsections. As the DTDL contents property is optional, the lack of contents in a DTDL Interface will be mapped in OPC UA with empty folders.

The DTDL comment property allows to define a comment for model authors. It has been mapped through the OPC UA Node named Comment, which is an optional property of the ObjectType DTDLInterfaceType, as clearly shown by Figure 4.

The DTDL schemas property represents the set of the descriptions of the data types in a digital twin interface. A full set of primitive data types are provided in DTDL, along with support for a variety of complex schemas in the forms of Arrays, Enums, Maps and Objects. In OPC UA the DataType NodeClass may be used to define the representation of the DTDL data types. As said in Section 3, OPC UA DataType may be Built-in, Enumeration or Structured; arrays of elements are also allowed. DTDL primitive data types may be easily mapped to the OPC UA built-in types, whilst DTDL complex schemas may be mapped to the OPC UA Enumeration, Structured and Array DataType. Proposals about this kind of mapping were considered outside the scope of this work. The DTDL schemas property is mapped into OPC UA through a set of OPC UA Nodes, each of which contains the information (i.e., the NodeId) of the OPC UA DataType mapping each single DTDL schema. As shown by Figure 4, the OPC UA FolderType Object called Schemas has been used to organize custom OPC UA PropertyType Nodes (called <Schema> in Figure 4), each of which contains, in the Value attribute, the NodeId of the OPC UA DataType mapping each DTDL schema exposed by the current interface.

The last mandatory folder present in Figure 4 is the Extends FolderType Object. It maps the DTDL extends property of the Interface Metamodel Class. In DTDL, Interfaces can inherit from multiple interfaces; for this reason, the DTDL extends property is used to maintain details of the set of interfaces each interface inherits from. Considering OPC UA domain, existing recommendations suggests using object composition instead of multiple inheritance. As explained in Section 3, the OPC UA feature based on AddIn and HasAddIn reference allows the creation of reusable components that may belong to many different ObjectTypes. This explains the use of HasAddIn reference in Figure 4, allowing the Extends folder to point to the DTDLInterfaceType Objects modelling the DTDL Interfaces the current interface inherits from.

### 5.2. Telemetry

The metamodel class Telemetry describes the data emitted by any digital twin, whether the data is a regular stream of sensor readings or a computed stream of data, such as occupancy, or an occasional error or information message. Telemetry does not store any data. This class has several properties shown by Table 5.

Due to the lack of data stored within a DTDL Telemetry element, it has been assumed to map it with a custom OPC UA ObjectType, called DTDLTelemetryType. Some of the attributes of the OPC UA Base NodeClass are used to represent a subset of the properties of the DTDL Telemetry class, as described by Table 6.

The same considerations accomplished for the properties @id, description and displayName seen for the Interface class are still valid and can be applied to the Telemetry class. The DTDL name property is defined as the “programming” name of the telemetry; mapping with the OPC UA BrowseName attribute seems suitable.

Considering the other DTDL properties shown by Table 5, the following considerations may be accomplished.

The DTDL @type property may be mapped as accomplished for the Interface class. In particular, for each OPC UA Object of OPC UA DTDLTelemetryType type used to represent the DTDL Telemetry, the @type property may be represented by the HasTypeDefinition Reference pointing to the OPC UA DTDLTelemetryType type.

**Figure 5:**
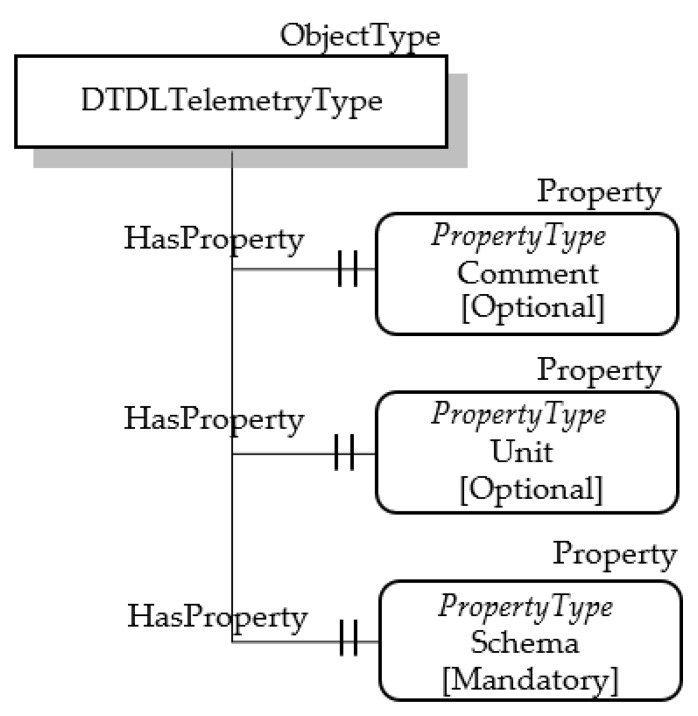
OPC UA DTDL TelemetryType.

The other DTDL properties present in Table 5 may be mapped by OPC UA Nodes, as shown in Figure 5.

The ObjectType DTDLTelemetryType has two optional OPC UA properties, Comment and Unit, mapping the DTDL properties comment (which allows to define a comment for model authors) and unit (which defines a semantic type of the Telemetry), respectively.

The DTDL schema property represents the data type of the Telemetry; OPC UA DataType seems suitable to represent this property, as said in the previous subsection. The proposed approach is to map the DTDL schema property with a custom OPC UA PropertyType Node, which contains information about the OPC UA DataType chosen to represent the DTDL data type. The attribute Value of the OPC UA PropertyType Node is used to contain this information. On the basis of this assumption, a custom OPC UA PropertyType Node has been considered for the DTDLTelemetryType ObjectType; Figure 5 shows this mandatory node called Schema. Its Value attribute will contain the NodeId of the OPC UA DataType mapping the current DTDL schema.

### 5.3. Property

The metamodel class Property allows to store values within a digital twin. These values can be read-only or have read and write states. For example, a device serial number may be a read-only value that can be read at any time; the desired temperature on a thermostat may be a read-write value that can be updated. This class has several properties, shown by Table 7.

As the DTDL Property class is used to store values, it seems that it may be represented very well by the OPC UA DataVariable Node. For this reason, it has been assumed to map the DTDL Property class with a custom OPC UA DataVariableType, called DTDLPropertyType, derived from the BaseDataVariableType.

Some of the basic attribute of the DTDLPropertyType may be used to represent properties of the Property class. Table 8 shows the proposed mapping of Property class properties with the attributes of OPC UA DTDLPropertyType. About the DTDL properties name, @id, description and displayName, the same considerations accomplished about the same properties seen for the two previous classes are still valid and can be applied to the Property class. The DTDL schema property represents the data type of the Property; as said before, OPC UA DataType seems suitable to represent this property. As a custom OPC UA DataVariableType has been considered to map the DTDL Property, the attribute DataType can be used to map the DTDL schema. About the writable property, it has been mapped to the WriteMask attribute as the semantic is exactly the same.

**Figure 6:**
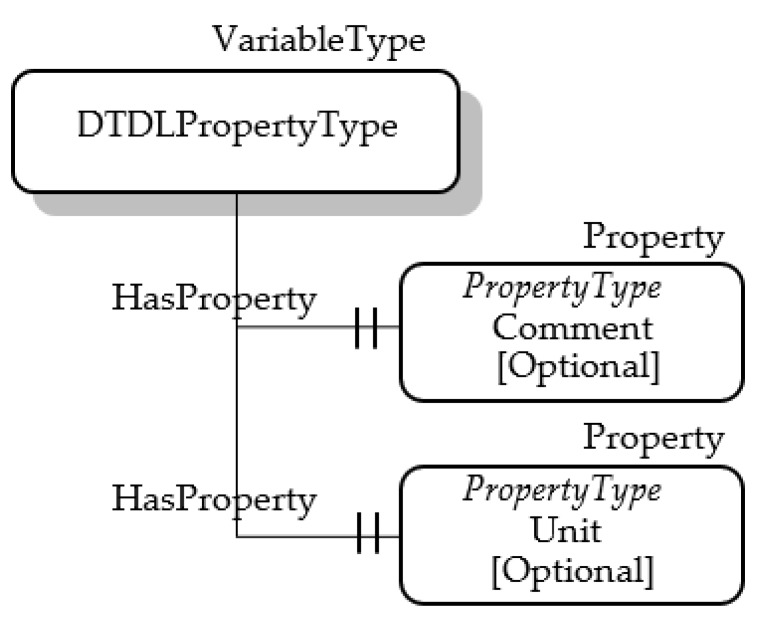
OPC UA DTDL PropertyType.

About the other properties, mapping of @type has been accomplished using the same approach described in the previous subsections. The other properties have been mapped, as shown by Figure 6; the DTDLPropertyType features two optional properties, Comment and Unit, mapping the DTDL properties comment and unit, respectively.

It is important to point out that the proposed modelling of the DTDL Property class with the custom DTDLPropertyType DataVariableType allows to make available the OPC UA attribute Value, which may be used to maintain the current value of the digital twin property. According to the WriteMask attribute, the value may be read-only, or it may be updated. This is very important to be pointed out, as this feature allows to fully map the capability of DTDL Property class to store values within a Digital Twin.

### 5.4. Command

The metamodel class DTDL Command allows to describe a function or operation that can be performed on digital twins. Table 9 shows the properties defined for this class.

It was assumed to represent the Command class through a custom OPC UA ObjectType called the DTDLCommandType. The reason of this mapping is due to the need to represent both properties and method; on account of what said about OPC UA, the only NodeClass suitable to represent complex structure is the ObjectType. The DTDLCommandType will inherit all the attributes of the OPC UA Base NodeClass. Some of these attributes are used to represent the properties of the Command class, as shown by Table 10. The considerations about mapping of the properties shown in the table, accomplished for the previous mappings, are still valid for the Command class.

About the other properties, mapping of @type has been accomplished using the same approach described for the DTDL classes seen before. In particular, it has been assumed that this property was represented by the HasTypeDefinition Reference pointing to the OPC UA DTDLCommandType ObjectType for each OPC UA Object used to represent a DTDL Command.

**Figure 7:**
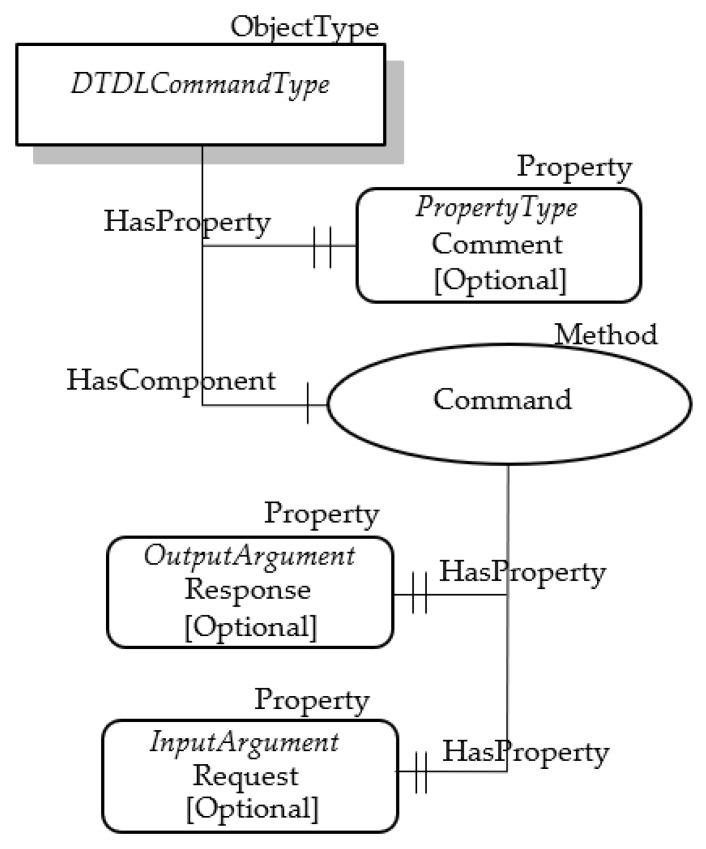
OPC UA DTDLCommandType.

Three properties of the Command class are still to be mapped into OPC UA, i.e., comment, request and response. Furthermore, the function or operation represented by the Command class must be modelled in OPC UA. In order to realize these mappings, the structure shown by Figure 7 has been considered. As shown, the DTDLCommandType ObjectType features a property and a method. The optional property called Comment models the comment property of the DTDL Command class. The OPC UA method called Command represents the function/operation modelled by the DTDL Command class; according to the OPC UA specifications, each method features optional input and output parameters. In this proposal, they are called Request and Response, respectively, and represents the relevant properties of the DTDL Command class.

### 5.5. Relationship

The metamodel class DTDL Relationship describes a link to another (separate) digital twin and enables graphs of digital twins to be created. The class features the properties shown by Table 11.

As accomplished for other DTDL metamodel classes, this class has been mapped using an OPC UA ObjectsType. A custom ObjectType has been defined and called DTDLRelationshipType. Table 12 shows the mapping of some of the Relationship class properties with the attributes of this OPC UA ObjectType. The same considerations about the mapping choices accomplished for the other previous classes may be applied for these properties.

**Figure 8:**
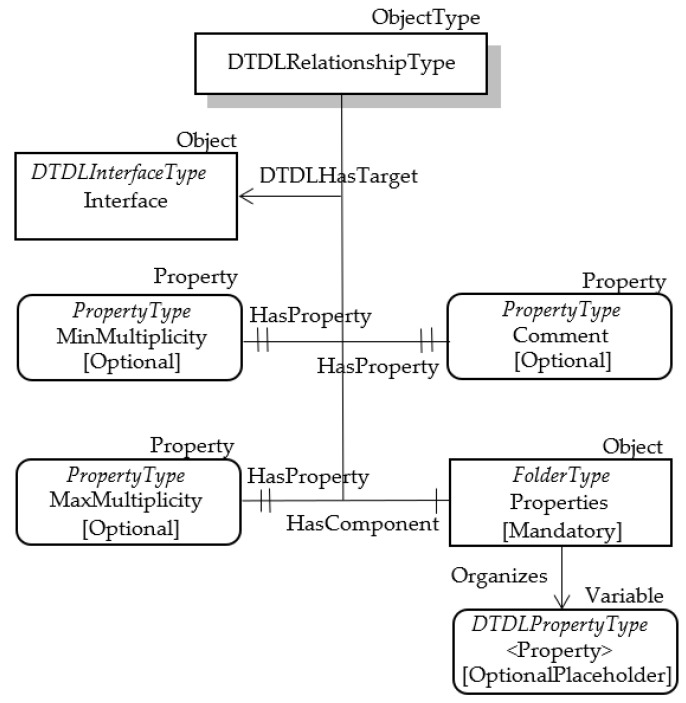
OPC UA DTDLRelationshipType.

About the other properties shown by Table 11, mapping of @type has been accomplished using the same approach described before, i.e., using the HasTypeDefinition Reference. The other properties were mapped using OPC UA Nodes, according to the schema shown by Figure 8.

It has been assumed that the DTDLRelationshipType ObjectType features three optional properties named Comment, MinMultiplicity and MaxMultiplicity. They model the DTDL properties comment, minMultiplicity and maxMultiplicity, respectively. The comment property is a comment for model authors, whilst minMultiplicity and maxMultiplicity represent the minimum and maximum multiplicity for the target of the relationship, respectively.

The DTDL property named properties represents the set of Property classes that define relationship-specific state; in other words, they define the main features of the relationship, if required. In order to represent this set, a folder has been considered in OPC UA; in Figure 8, it is called Properties. This folder will contain OPC UA Nodes, each modelling a Property class; Figure 8 shows that the folder Properties organizes OPC UA Property Nodes belonging to DTDLPropertyType. As the DTDL properties is optional, the Modelling Rule relevant to each OPC UA Property Node is an OptionalPlaceholder.

**Figure 9:**
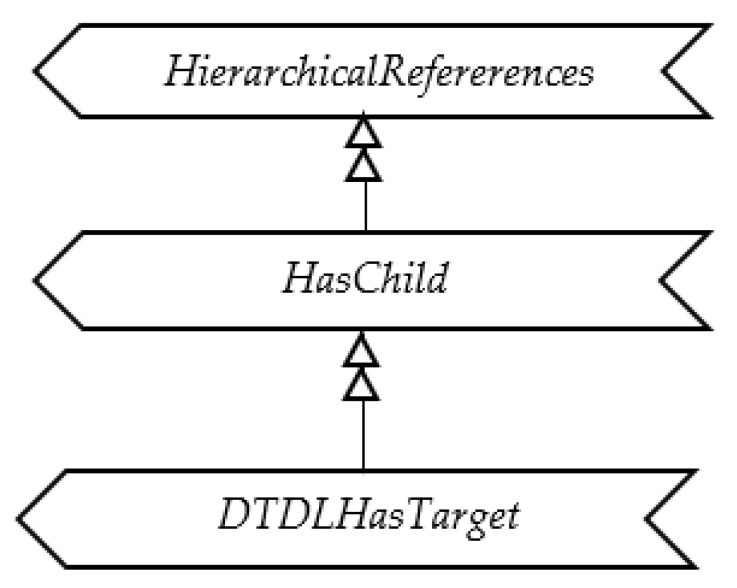
OPC UA DTDLHasTarget Reference.

The last DTDL property to be mapped is the target, representing the link to a DTDL Interface. In OPC UA, the links between Nodes are realized using References, as explained in Section 3; for this reason, the DTDL target was mapped into OPC UA through a Reference pointing to the OPC UA Node modelling the Interface to which the Relationship refers. A custom Reference, called DTDLHasTarget, has been defined as a subtype of HasChild (that is, in turn, a subtype of HierarchicalReference), as shown by Figure 9. Figure 8 shows that the DTDLHasTarget reference allows an Object of DTDLRelationshipType type to point to a DTDLInterfaceType Node, modelling a DTDL Interface. It is very important to point out that the DTDLInterfaceType Object shown in Figure 8, with the generic name Interface, must be an Object already existing and representing a DTDL Interface as said before.

### 5.6. Component

The metamodel class Component enables interfaces to be composed of other interfaces. Components are different from Relationships, because they describe contents that are directly part of the interface, whilst a relationship describes a link between two interfaces. Table 13 shows the properties defined for this class.

A custom OPC UA ObjectType NodeClass has been used to represent the Component class; it was named DTDLComponentType ObjectType. Table 14 shows the mapping of some properties of the DTDL Component class with the attributes of OPC UA DTDLComponentType.

**Figure 10:**
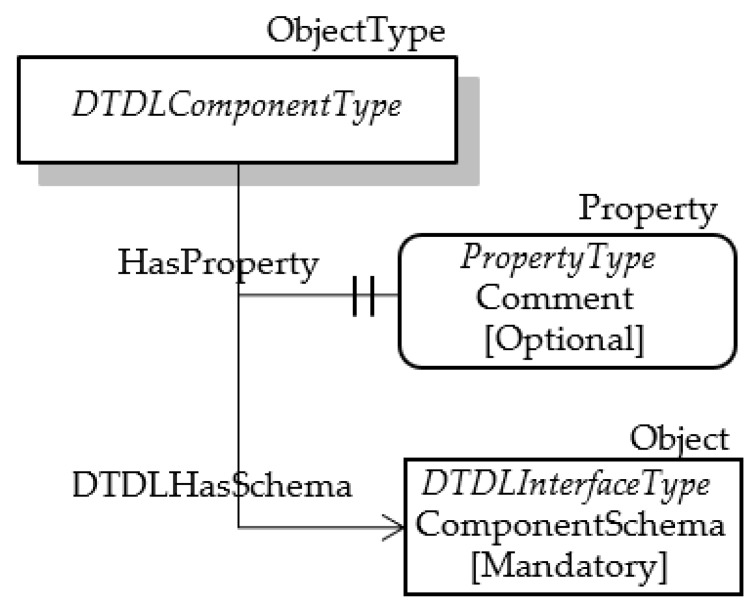
OPC UA DTDLComponentType.

The property @type has been modelled using the HasTypeDefinition Reference, as explained in the previous subsections. The properties comments and schema have been mapped using OPC UA Nodes, as shown by Figure 10. The DTDLComponentType ObjectType has an optional property called Comment; it models the DTDL property comment. The other DTDL property to be mapped is the schema, which defines the DTDL Interface of the component. As the DTDL Interface has been modelled in OPC UA by an Object of the DTDLInterfaceType type, Figure 10 shows the presence of a mandatory Object of this type. In order to allow the DTDLComponentType Object to point to the DTDLInterfaceType Object, a custom Reference has been defined called DTDLHasSchema, as shown by Figure 10. The DTDLHasTarget Reference has been defined as a subtype of the HasComponent Reference, which is a Hierarchical Reference, as stated in Section 3.

## 6. Implementation and Validation

The custom OPC UA Information Model presented in the previous section has been implemented by the authors and the software implementation is freely available at the GitHub repository [41]. In particular, the repository maintains the “dtdl_opcua_information_models_mapping.tt2pro” file; the reader may use free software tools such as UaModeler from Unified Automation [42] to import this file and explore the custom OPC UA NodeClasses implemented and described in the previous sections. The repository [41] maintains also a xml file that can be used to realize an OPC UA Server by developing a custom program; OPC UA Foundation provides a standard syntax, called NodeSet2, containing the description in xml of the (standard or custom) OPC UA information model to create and populate an AddressSpace inside a server. In particular, the xml available in [41] contains the custom OPC UA NodeClasses described in Section 5. The process of importing the xml file in a program depends on the OPC UA SDK used to create the OPC UA Server.

The implementation of the custom OPC UA information model presented in this paper, allowed the authors to validate the proposal. Several case studies were considered, and real mappings were realized and tested. In order to give a clear idea about the procedure used to validate the proposal, in the following subsections, details about the different steps followed in the validation procedure will be given. Details will refer to a single case study; a very simple example has been considered in order to be easily read and understood.

### 6.1. Case Study

It has been assumed to take into consideration a Digital Twin of a building, modelling each component and, in particular, each room. In particular, the DT of a room will be considered in this case study. A basic model of a Room has been defined, and its extension, called MeetingRoom, has been considered. The MeetingRoom model includes all the properties of the Room model, adding other ones.

**Figure 11:**
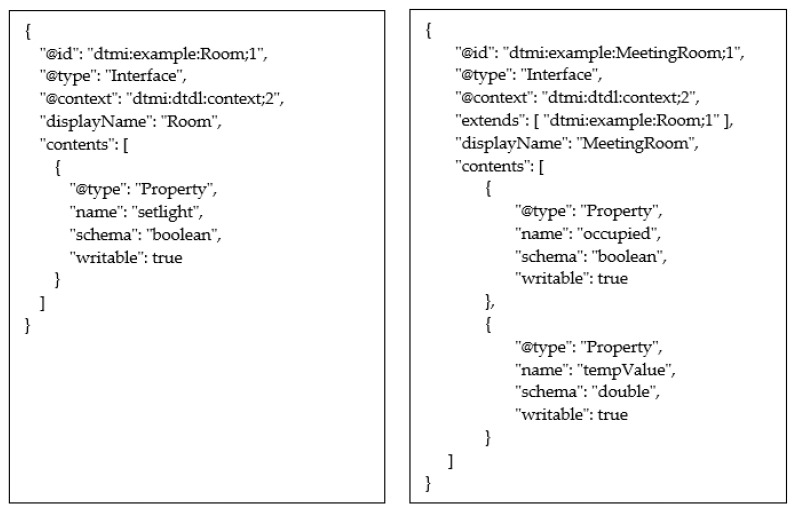
Example of the DTDL Interface: model of a Room (**left**) and model of a MeetingRoom (**right**).

The DTDL Interface models shown by Figure 11 are considered. As it can be seen, the interface called “MeetingRoom” is shown on the right; it features a contents property made up by two Property elements (i.e., “occupied” and “tempValue”). This interface extends a simpler interface called “Room”, shown in the same Figure 11 on the left; the DTDL Interface “Room” is made up by only one Property (i.e., “setlight”).

### 6.2. Digital Twin Instance in Microsoft Azure Platform

**Figure 12:**
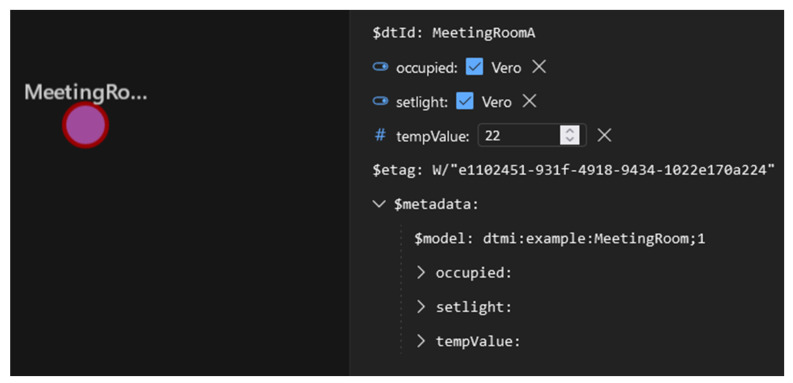
Graphical Representation of Digital Twin MeetingRoomA in Azure DT Explorer.

The DTDL “MeetingRoom” model shown by Figure 11 was implemented inside Microsoft Azure Digital Twin platform available in [43]. A particular tool named “Azure Digital Twin Explorer” is available in this platform to import DTDL models and to create instance of Digital Twins based on DTDL. The Room and MeetingRoom DTDL models were uploaded in this tool, and a single instance of the “MeetingRoom” Interface has been created; the instance was named “MeetingRoomA”. Figure 12 shows the graphical representation of this instance inside the “Azure Digital Twin Explorer” tool.

As it can be seen from Figure 12, the Digital Twin instance is graphically represented by a circle, and all the main properties of this instance are shown on the right side. First of all, the identifier of the Digital Twin instance is clearly specified on the top; it is “MeetingRoomA”, as said before. The figure shows the three properties occupied, setlight and tempValue featured by the DTDL MeetingRoom model. The actual values of these properties are also shown (e.g., value 22 for the tempValue). These values were assigned by the authors through the explorer tool.

### 6.3. Definition of an OPC UA Server

According to Figure 1, the next step of the proposed mapping is the definition of an OPC UA Server able to map each Digital Twin instance into the OPC UA domain. In our simple case study, the Digital Twin instance is “MeetingRoomA”, shown by Figure 12. This instance and all the related information must be mapped into the OPC UA domain.

OPC UA Server was implemented in NodeJS using NodeOPCUA [44], which is an OPC UA SDK, making available the OPC UA communication stack written in TypeScript for NodeJS; this stack is needed in order to use the OPC UA standard services, including those needed to access the AddressSpace. The Azure SDK for NodeJS [45] available on GitHub has also been used; this software realizes the communication stack needed to the access to the Digital Twin instances through a NodeJS program.

The realization of the OPC UA Server must include the implementation of the custom AddressSpace proposed in this paper and the code of the suitable procedures to populate this AddressSpace with real values.

**Figure 13:**
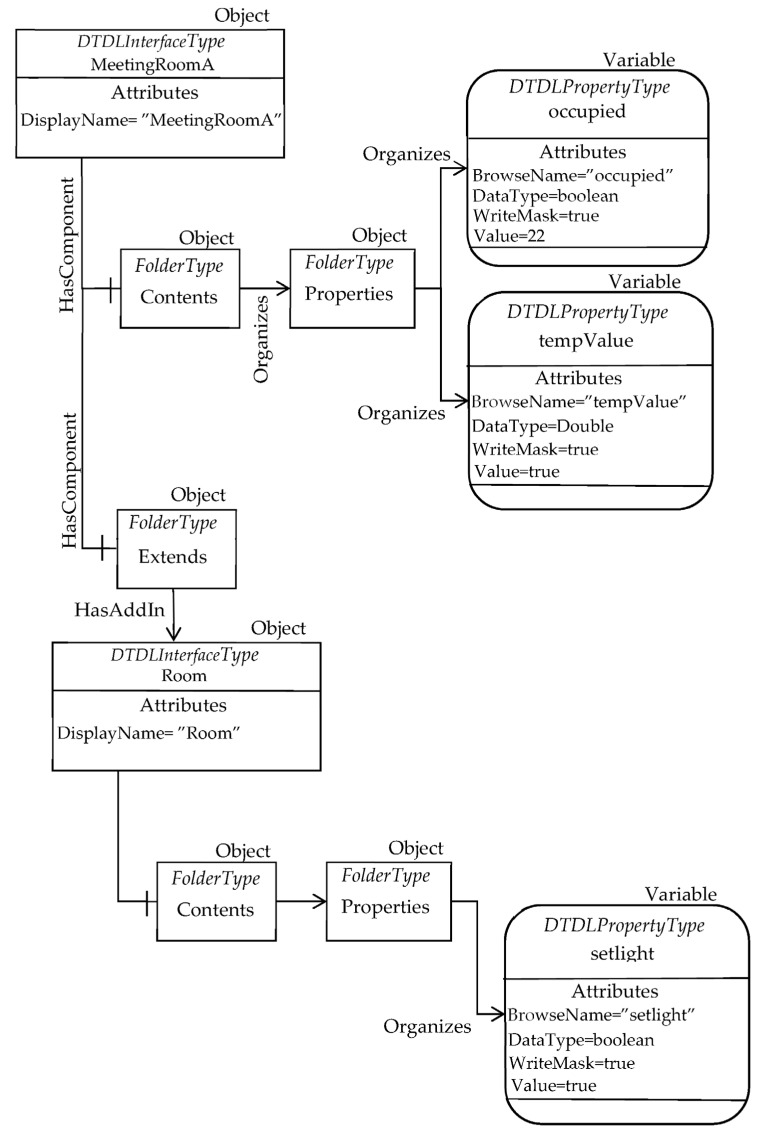
Example of mapping using the OPC UA DTDL InterfaceType.

Applying the mapping solution presented in Section 5, the OPC UA AdressSpace mapping the “MeetingRoomA” instance may be achieved. Figure 13 shows the OPC UA Nodes realizing this mapping.

In particular, the OPC UA Object “MeetingRoomA” is an instance of the DTDLInterfaceType ObjectType, representing the DTDL Interface model. It features a component (pointed by the HasComponent reference) made by the mandatory folder Contents; this folder organizes the folder Properties, organizing two OPC UA DataVariable Nodes instances of the DTDLPropertyType type. They model the two DTDL properties “occupied” and “tempValue”. Figure 13 points out the main attributes of these Objects, including the DataType, the WriteMask and the Value. It is important to point out that the contents of the Value attribute are set exactly to the same values shown in Figure 12, i.e., true for occupied and 22 for tempValue.

The OPC UA Object “MeetingRoomA” features another component made up by the mandatory folder Extends. As explained in Section 5.1, this folder is used to map the extends property of the Interface class. The HasAddIn reference in Figure 13 allows the Extends folder to point to the DTDLInterfaceType Object modelling the DTDL Interfaces the current interface inherits from, i.e., the Interface named “Room”, according to the DTDL description shown by Figure 11 on the left.

The OPC UA DTDLInterfaceType Object “Room” represents the Interface with name “Room”, and it features only one OPC UA DataVariable Node organized by the Properties folder. This Node represents the DTDL property named “setlight”, shown in Figure 11. Again, several attributes are shown, including the Value, which contains the same value shown for the Digital Twin instance in Figure 12.

It is important to point out that the other mandatory folders of the DTDLInterfaceType ObjectType (see Figure 4) are not represented in Figure 13 only for space reasons. According to the content of the DTDL Digital Twin given by Figure 11, these folders are empty, as no DTDL entities are present to be organized by the lacking mandatory folders.

**Figure 14:**
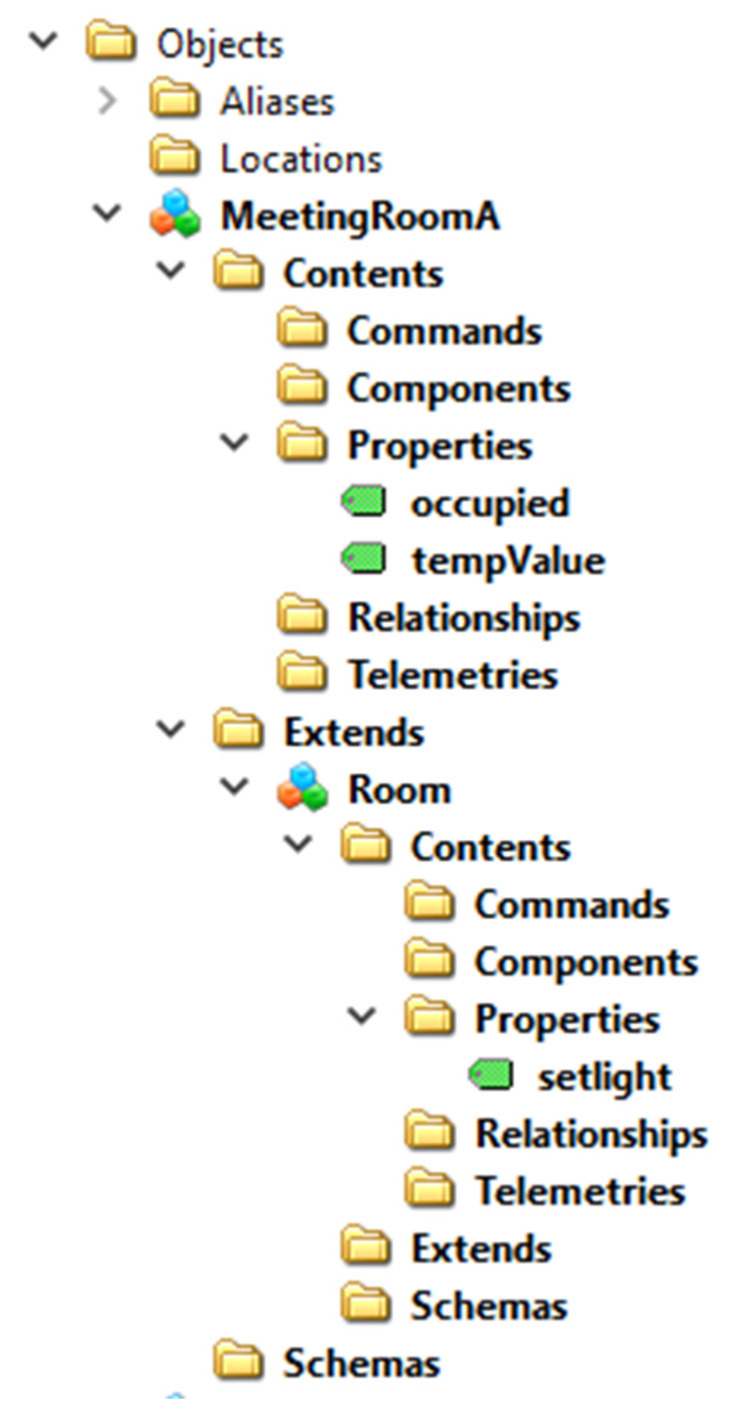
OPC UA Nodes representing MeetingRoomA in the OPC UA domain.

The OPC UA AddressSpace shown by Figure 13 was implemented using the UaModeler [42] tool. Figure 14 shows the graphical representations of the OPC UA Nodes that have been created using the UaModeler tool.

As said before, the Digital Twin instance “MeetingRoomA” features three properties (i.e., occupied, tempValue and setlight) whose value changes over the time. Changes in the DT instance are generally due to updates of the real system for which the Digital Twin realizes a digital replica. Using the “Azure Digital Twin Explorer”, these values may be changed by the user in order to simulate the updates from the real system, as accomplished by the authors during the validation procedure (Figure 12 shows the actual values of these properties, which were assigned by the authors through the explorer tool).

It is clear that the information maintained by the Digital Twin instance and by the OPC UA Server must be consistent. This means that each change in a property of the Digital Twin instance must be updated into the OPC UA Server and vice versa. For this reason, realization of the OPC UA AddressSpace as shown by Figure 14 in the server is not enough, but a custom program is needed to be implemented inside the server to realize this update in automatic fashion. Each time information changes inside the Digital Twin instance, the same change must be reflected in the OPC UA Server and vice versa.

Only the most important pieces of code relevant to the updating of the information from Digital Twin will be presented in the remainder of this section.

**Figure 15:**
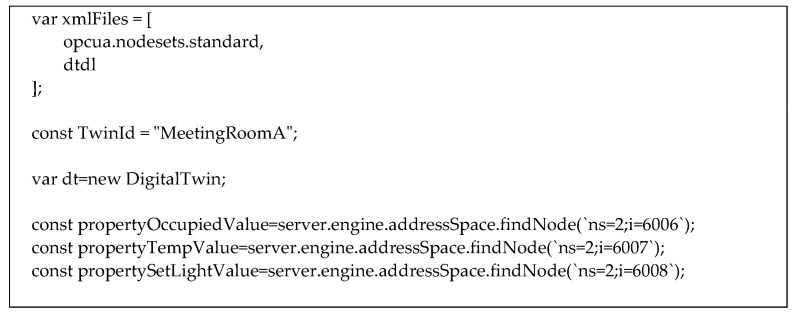
Initializations needed in the OPC UA Server in NodeJS.

Figure 15 shows the code relevant to the main initializations needed in the server.

At the beginning, the OPC UA AddressSpace to be used in the server must be created; this is accomplished through variable “xmlFiles” that contain xml files written according to the NodeSet2 format. In the figure, the “opcua.nodesets.standard” file includes the standard information model, whilst the “dtdl” file includes the custom information model presented in Section 5, plus the set of nodes shown by Figure 13 and Figure 14.

A constant containing the name of the Digital Twin instance “MeetingRoomA” is defined. Creation of an instance of the Digital Twin class (i.e., “dt” in Figure 15) is required to manage authentication and communication with the Microsoft Azure Digital Twin instance.

Figure 14 shows that three OPC UA Nodes are present in the OPC UA AddressSpace of the server representing the three properties of the Digital Twin instance “MeetingRoomA” (i.e., occupied, tempValue and setlight). In this example, it has been assumed that these three nodes feature the NodeIds (‘ns=2;i=6006’), (‘ns=2;i=6007’) and (‘ns=2;i=6008’), respectively. The function “server.engine.addressSpace.findNode()” allows to find the node of interest within the OPC UA Server AddressSpace, passing the relevant NodeId (made up by a namespaceindex and identifier); it returns an UaVariable relevant to the contents of the node specified. In this example, the variable “propertyTempValue” is relevant to the node with NodeId (‘ns=2;i=6007’); each change in the Node is accomplished also on the variable and vice versa.

**Figure 16:**
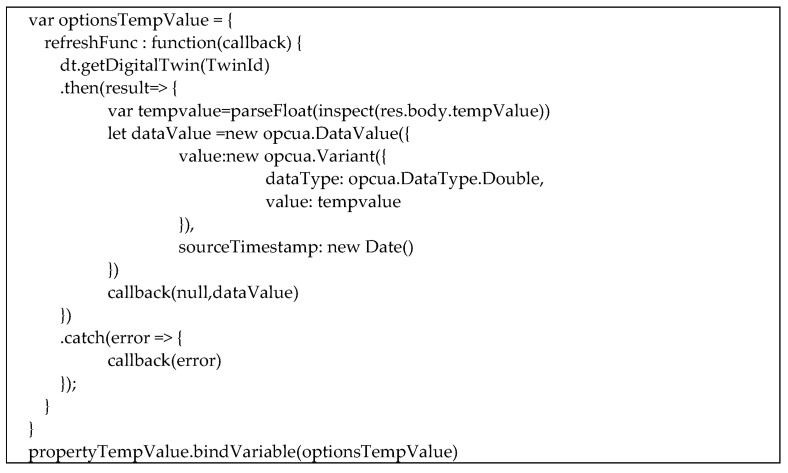
Automatic update of the tempValue property.

Figure 16 shows another piece of code implemented in the OPC UA Server relevant only to the automatic updated of the property tempValue of the DT instance “MeetingRoomA”.

As shown by Figure 16, the function “dt.getDigitalTwin()” is used; it invokes a service provided by Azure Microsoft allowing to access to any details of any digital twin instance specified as an argument of the call. Considering the DT instance shown by Figure 12, the call of this function allows to achieve several pieces information, among which are $dtId, $metadata, $model and tempValue. In particular, here, the authors are interested only to the tempValue property; for this reason, the variable tempvalue in Figure 16 is set to the current value of tempValue property from the Digital Twin instance.

In order to realize an automatic update of each value of the property tempValue, the asynchronous method “refreshFunc” shown by Figure 16 is used; the method is in charge to invoke the “dt.getDigitalTwin(TwinId)”, and when the information from the DT instance is received, the value of the tempValue property is delivered to the server by the “callback” function shown by Figure 16. As it can be seen, the callback features several arguments, among which is a DataValue with the updated value of tempValue.

The last code shown by Figure 16, “propertyTempValue.bindVariable(optionsTempValue)”, is very important, as it associates the “refreshFunc” with the OPC UA Node with NodeId (‘ns=2;i=6007’), i.e., the propertyTempValue variable. The consequence of this code is that, every time a client desires to read the current value of the OPC UA Node with NodeId (‘ns=2;i=6007’), the “refreshFunc” is invoked, and the last value of the tempValue property of the DT is updated in the OPC UA Server.

### 6.4. Verification of Consistency

The last step followed in the validation procedure adopted by the author was the execution of several tests aimed to verify the consistency between information maintained by Digital Twin instance and OPC UA AddressSpace. Considering the simple case study presented here, several changes in the properties of the DT instance “MeetingRoomA” were performed, verifying that the consistent changes occurred in the OPC UA Server, accessing the information by OPC UA client applications.

## 7. Discussions and Conclusions

The paper introduced a solution of mapping from DTDL to the OPC UA Information Model. The proposal allows to represent each DTDL element into a corresponding OPC UA element. This allows to enable the interoperability of DTDL-based Digital Twin. Through the solution proposed in the paper, information maintained by a DTDL-based Digital Twin can be published by an OPC UA server that makes this information available to any OPC UA-compatible device (acting as OPC UA client and/or OPC UA subscriber). The authors have presented an overview of the current state-of-the-art, pointing out the originality of their work. Furthermore, a software implementation has been presented; it is freely available on the GitHub repository. Implementation was allowed to validate the proposed approach.

The authors believe that the proposed mapping accomplishes the goals set by the fourth industrial revolution, creating systems and services increasingly flexible, interoperable and innovative, improving the quality and efficiency of industrial production, by using Digital Twins. They believe that the proposed solution opens up a lot of opportunities in the interoperability between applications based on Microsoft Azure Digital Twin and the OPC UA domain.

## Figures and Tables

**Table 1:** Graphical representation of some OPC UA Nodes.

OPC UA Node	Standard Graphical Representation
ObjectType	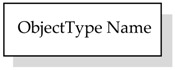
VariableType	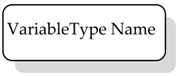
Object	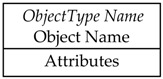
Variable	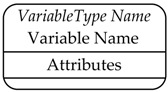
ModellingRule Object & HasModellingRule Reference Note: the ModellingRule Object and the relevant HasModellingRule Reference are not represented; only the InstanceDeclaration is given, as shown on the right (the kind of ModellingRule is specified inside square brackets)	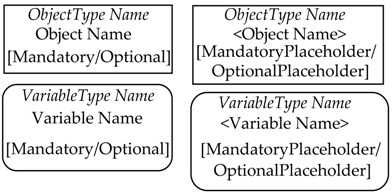
Method	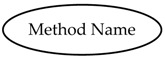
Reference	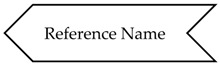

**Table 2:** Graphical representation of some OPC UA References.

OPC UA Reference	Standard Graphical Representation
HasProperty	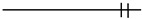
HasComponent	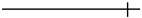
Hierarchical (e.g., Organizes, HasAddIn)	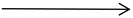
HasTypeDefinition	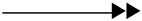
HasSubtype	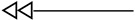

**Table 3:** Properties of Interface class.

Property	Mandatory/Optional
@type	mandatory
@id	mandatory
@context	mandatory
comment	optional
contents	optional
description	optional
displayName	optional
extends	optional
schemas	optional

**Table 4:** Mapping of Interface class properties into OPC UA DTDLInterfaceType attributes.

Interface Class Properties	DTDLInterfaceType Attributes
@id	NodeId
displayName	DisplayName
description	Description

**Table 5:** Properties of the Telemetry class.

Property	Mandatory/Optional
@type	mandatory
name	mandatory
schema	mandatory
@id	optional
comment	optional
description	optional
displayName	optional
unit	optional

**Table 6:** Mapping of properties of the Telemetry class into OPC UA DTDLTelemetryType attributes.

Telemetry Class Properties	OPC UA DTDLTelemetryType Attributes
name	BrowseName
@id	NodeId
description	Description
displayName	DisplayName

**Table 7:** Properties of the Property class.

Property	Mandatory/Optional
@type	mandatory
name	mandatory
schema	mandatory
@id	optional
comment	optional
description	optional
displayName	optional
unit	optional
writable	optional

**Table 8:** Mapping of properties of the Property class into OPC UA DTDL PropertyType attributes.

Property Class Properties	OPC UA DTDL PropertyType Attributes
name	BrowseName
schema	DataType
@id	NodeId
description	Description
displayName	DisplayName
writable	WriteMask

**Table 9:** Properties of the Command class.

Property	Mandatory/Optional
@type	mandatory
name	mandatory
@id	optional
comment	optional
description	optional
displayName	optional
request	optional
response	optional

**Table 10:** Mapping of properties of the Command class into the OPC UA DTDLCommandType attributes.

Command Class Properties	OPC UA DTDLCommandType Attributes
name	BrowseName
@id	NodeId
description	Description
displayName	DisplayName

**Table 11:** Properties of the Relationship class.

Properties	Mandatory/Optional
@type	mandatory
name	mandatory
@id	optional
comment	optional
description	optional
displayName	optional
maxMultiplicity	optional
minMultiplicity	optional
properties	optional
target	optional
writable	optional

**Table 12:** Mapping of properties of the Relationship class into the OPC UA DTDLRelationshipType attributes.

Relationship Class Properties	OPC UA DTDLRelationshipType Attributes
name	BrowseName
@id	NodeId
description	Description
displayName	DisplayName
writable	WriteMask

**Table 13:** Properties of the Component class.

Property	Mandatory/Optional
@type	mandatory
name	mandatory
schema	mandatory
@id	optional
comment	optional
description	optional
displayName	optional

**Table 14:** Mapping of properties of the Component class into the OPC UA DTDLComponentType attributes.

Component Class Properties	OPC UA DTDLComponentType Attributes
name	BrowseName
@id	NodeId
description	Description
displayName	DisplayName

## Data Availability

Not applicable.
